# Blue Eyes Help Men Reduce the Cost of Cuckoldry

**DOI:** 10.1007/s10508-021-02120-7

**Published:** 2021-09-27

**Authors:** Paola Bressan

**Affiliations:** Dipartimento di Psicologia Generale, University of Padova, 35131 Padova, Italy

**Keywords:** Eye color, Paternity confidence, Paternal rejection, Assortative mating, Mate choice

## Abstract

Men with light eyes lack the dominant gene allele that codes for dark-brown eyes. Pairing with a woman who lacks the same allele must increase paternity confidence in these men, because any children with dark eyes would be extremely unlikely to have been fathered by them. This notion implies that men with light (blue or green) eyes should (1) prefer light-eyed women, especially in a long-term context, and (2) feel more threatened by light-eyed than by dark-eyed rivals. Yet because choosiness is costly and paternity concerns are entirely driven by the prospect of paternal investment, any such inclinations would be adaptive only in men who expect to invest in their children. Here I test these ideas using the data of over 1000 men who rated the facial attractiveness of potential partners, and the threat of potential rivals, whose eye color had been manipulated. Light-eyed men liked light-eyed women better (particularly as long-term companions), and feared light-eyed rivals more, than did dark-eyed men. An exploratory analysis showed that these large, robust effects disappeared in men who had felt rejected by their fathers while growing up—suggesting that such men are not expecting to invest in their own children either.

It is a wise father that knows his own child.

— William Shakespeare,* The Merchant of Venice, Act 2 Scene 2*

## Introduction

Males can never be certain they have fathered their mates’ offspring. What they can do is adjust parental care to the probability of being genetically related to them (Trivers, 1972). There is ample evidence that paternal effort is reduced in response to lowered likelihood of paternity in several species of birds, such as dunnocks and swallows (Davies & Quinn, 1992; Møller, 1988), and in some insects, for example in beetles (Hunt & Simmons, 2002).

In our species, too, paternal investment is strongly related to paternity confidence (Apicella & Marlowe, 2004; Fox & Bruce, 2001), and paternity confidence is strongly related to the probability of paternity. A survey of published estimates shows that in men with high paternity confidence the median non-paternity rate is below 2%, whereas in men with low paternity confidence it jumps to about 30% (Anderson, 2006). This suggests that men can detect the likelihood of extra-pair paternity *and* that this ability is highly imperfect.

To estimate their certainty of paternity, animals appear to use behavioral rules of thumb—for example, the number of copulations with their mate (Davies & Quinn, 1992) or by their mate with other males (Møller, 1988), or the frequency of their own encounters with sneaking males (Hunt & Simmons, 2002). The strategy that might look like the best—labeling one’s progeny with some distinctive, recognizable, heritable paternal badge—seems to be avoided. As it happens, direct offspring recognition has never been demonstrated (e.g., Kempenaers, 1996), suggesting that birds and beetles do not mark their offspring—although it seems that, in principle, they could label them with specific plumage or elytra cues.

Men do not appear to mark their offspring either, as suggested by different lines of evidence. First, paternal resemblance is no stronger than maternal resemblance. Children are matched to one parent as (un)reliably as to the other (Brédart & French, 1999); infants’ parental resemblance to one parent relative to the other is a Gaussian curve, with most individuals resembling mother and father about equally (Bressan & Grassi, 2004). Second, parental resemblance develops over time, rather than emerging at birth as it should do if it had evolved for the purpose of rejecting adulterine offspring: the same children are matched far more accurately to their true parents at 16 than at 1 year of age (Bressan & Dal Pos, 2012). Third, parental resemblance in very young children is quite poor. When judges are not informed about genetic relatedness, nearly 20% of 1-year-olds receive a parental resemblance rating of zero on a 0–10 scale, as opposed to less than 5% of 8-year-olds (Bressan & Grassi, 2004; Bressan & Dal Martello, 2002); and the probability of selecting a newborn’s correct parent (out of three potential ones) is, at best, 1.2 times higher than chance (McLain et al., 2000).

Yet, men do use children’s resemblance to themselves as a cue of genetic relatedness (Alexander, 1974). They invest more in children who resemble them more (e.g., Alvergne et al., 2009; Apicella & Marlowe, 2004). It may be argued that this is indeed why babies do not resemble their biological fathers. By being able to reject adulterine children, fathers who put their own stamp on their offspring do gain an evolutionary benefit. However, this gain is exactly counterbalanced by the evolutionary cost they pay by having their own extra-pair children rejected by other men (Bressan, 2002). Thus, forms of progeny identification based on indirect cues (such as lack of potential rivals) will be likely to evolve; preferential investment in those children who happen to resemble the investor will be likely to evolve; but above-chance offspring marking will not.

Some heritable traits come in both a recessive, “neutral” variant and a dominant, “marked” one. Now, a man who carries recessive alleles for a certain trait could in principle increase his paternity confidence simply by choosing, as a partner whose offspring will be the recipient of paternal investment, a woman who is also a recessive carrier of that trait. Any offspring presenting the dominant version of it will be unlikely to have been fathered by this man. An excellent example of such a trait is eye color, a highly heritable (98%: Balaresque & King, 2016) and visually salient feature. Eye color depends on the number and size of melanin particles in the layers of the iris: more melanin, darker eyes. But although the amount of melanin is determined by multiple genes, an important one serves as an on–off switch. This gene sits on chromosome 15 and has two alleles, one dominant and one recessive. The dominant, ancestral allele stimulates melanin production in the eye and, by making the eyes dark brown, masks the color that would be displayed otherwise. The recessive allele reduces melanin production fivefold (Duffy, 2015), allowing the eyes to appear any shade of blue, grey, green, or hazel (depending on other genes and modifiers), but not dark brown. If neither parent carries the dominant allele, thus, a child is extremely unlikely to have dark eyes. This description simplifies the underlying genetics considerably, but without undermining the argument: indeed, a single variation in the *HERC2* gene (rs12913832) permits to predict with high accuracy whether someone’s eyes are brown or blue (Duffy, 2015; Eiberg et al., 2008; Liu et al., 2013; Sturm et al., 2008).

Therefore, light-eyed men who prefer light eyes—and choose a light-eyed partner—will enjoy increased paternity confidence, because odds are that any dark-eyed child is the result of a liaison of their mate with another man. I am not suggesting, of course, that presumed fathers would be conscious of that. Light-eyed men who prefer light eyes would simply be more likely to invest in their own (rather than someone else’s) genes than would light-eyed men with the opposite preference, or no preference. This leads to the prediction that, other things being equal, men with light eyes should be especially attracted to women with light eyes. This has been shown to be the case in a study with 44 male participants who were asked to rate, from photographs, the attractiveness of faces with blue or brown eyes (Laeng et al., 2006). For each face, a digitally manipulated copy was produced with the alternative eye color. The 22 men with blue eyes preferred female faces with blue eyes as opposed to brown ones; the 22 men with brown eyes had no preferences. However, the blue- and brown-eyed versions of each face were never both presented to the same participant, let alone compared directly; also, hazel eyes were included and lumped together with brown ones, although hazel-eyed people typically carry one “blue” allele, sometimes two (Duffy, 2015). Such choices may have made the task more natural but would have introduced a lot of noise too; and noise, especially in samples as small as this, can create spurious effects.

Here I not only check, on a much larger sample and with a cleaner, more sensitive method, whether the effect truly exists, but I also test its evolutionary explanation. Two clear predictions stem directly from it. First, the preference of light-eyed men for light-eyed women should be adaptive only in men who are looking for a woman whose children they would expect to invest in. In most societies, a child’s arrival has the potential to invite investment from the alleged father whatever the context in which conception has occurred. Such an event can in fact catalyze the transformation of a casual, short-term relationship into a committed, long-term one. Thus, information on whether he has actually fathered this child must be important to a man at all times. Yet paternity confidence should be a special concern when a prospective partner’s qualities are explicitly evaluated for a long-term relationship: hence, this is the context in which the choices of light- and dark-eyed men should differ most. Second, light-eyed men should feel more threatened by rivals who are also light-eyed—because these are precisely the men whose children would, based on eye color, be impossible to identify as adulterine.

I tested the first prediction on the data of over one thousand men who were asked to rate, from face photographs, the attractiveness of light- and dark-eyed women as potential short- or long-term partners (analyses of a portion of these data, unrelated to the hypotheses examined here, have been presented in Bressan, 2020b). I tested the second prediction on the data of all the men in the sample who reported being partnered. These men were shown the photos of light- and dark-eyed potential rivals too, and rated how jealous of them they would feel if they suspected that their current partner was being unfaithful.

## Method

### Participants

The total sample included 1440 men (median age = 23 years, range = 18–73 years). With very few exceptions, participants were Italian; they were mostly recruited via links posted on Italian universities’ online social networks and other social media, such as Facebook groups. Men who self-identified as heterosexual or bisexual with a preference for female partners were directed to the current study, whereas men who self-identified as homosexuals or bisexual with a preference for male partners were directed to a separate one. The analyses presented here include all participants whose eyes were either blue or green (light-eyed men: *N* = 432) and either brown or dark brown (dark-eyed men: *N* = 805), for a total of 1237 individuals; of these, 621 reported currently having a partner. Data were collected in accordance with guidelines approved by the local Psychological Research Ethics Committee; all participants gave informed consent.

### Materials and Procedure

**Fig. 1:**
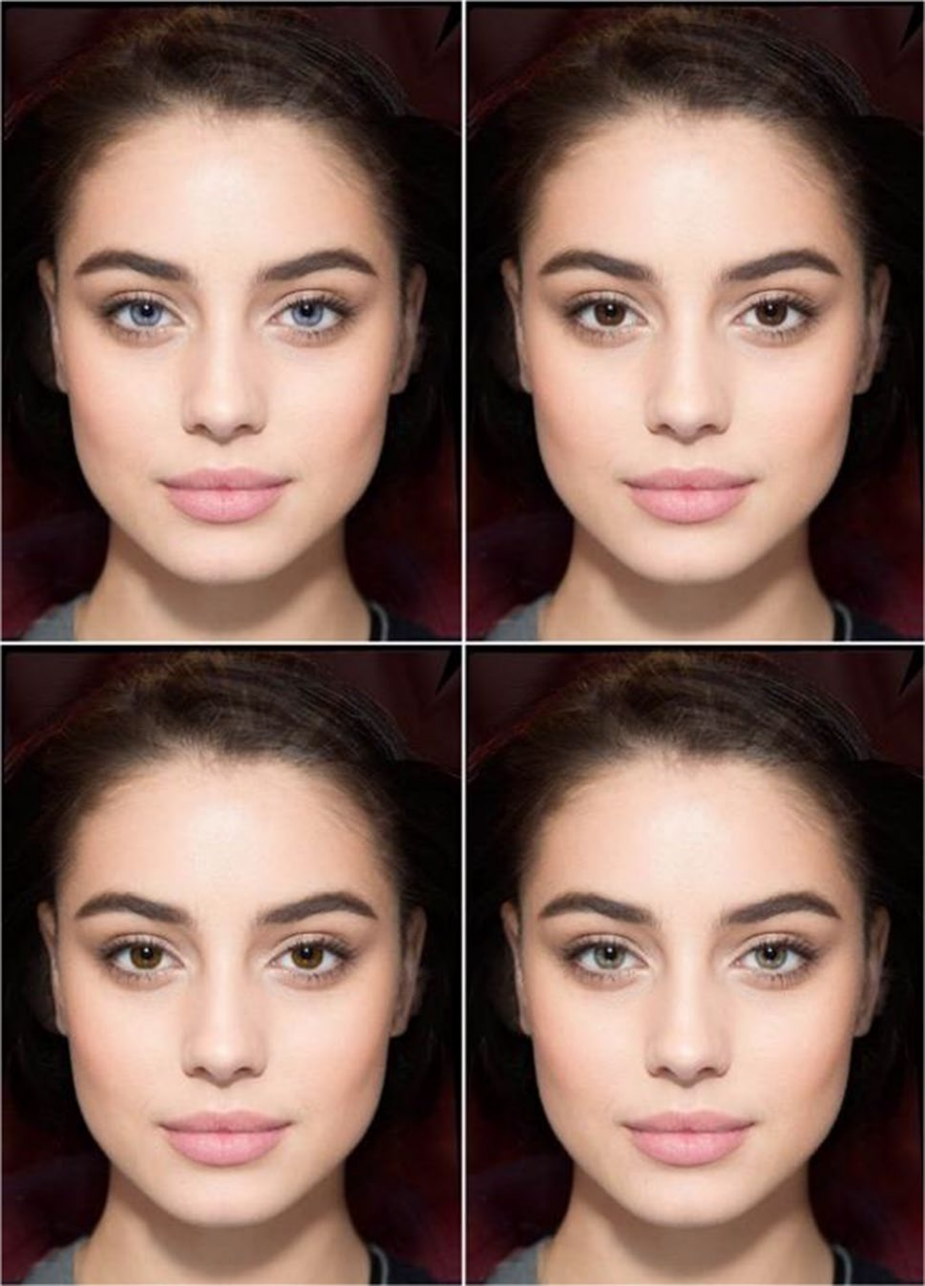
Example stimuli. Participants saw pairs of female faces differing only in eye color: light (blue or green) vs dark (brown or dark brown). Top: blue/dark brown pair. Bottom: brown/green pair. The face depicted here has been created digitally for purposes of illustration (Bressan, 2020a); the study showed photographs of real women (Color figure online)

The professional photo-retouching software PortraitPro was used to modify 10 facial photographs of attractive young women of European ancestry, so as to produce four versions of each face differing only in eye color. These were shown in pairs (Fig. 1): blue/dark brown (blue on the left) and brown/green (green on the right). For each face, the blue/dark brown pair was presented to half of the participants and the brown/green pair to the other half; everybody saw five blue/dark brown and five brown/green combinations, so that the side on which light and dark eyes appeared was automatically counterbalanced both within and between subjects. The 10 photo pairs were shown once in a short-term and once in a long-term relationship context, with context order counterbalanced between participants (see Bressan, 2021).

Participants were asked three questions. The first was, “If you were looking for a long- (short-) term relationship, which of these two people would you prefer?” Possible responses were “the one on the left” and “the one on the right.” The other questions were, “For a long- (short-) term relationship, how attractive do you find the person on the left?” and “For a long- (short-) term relationship, how attractive do you find the person on the right?”, each followed by a 0–10 scale going from “not at all attractive” to “very attractive.” Long-term and short-term relationships were defined as follows: “By a long-term relationship, we mean someone you may consider leaving your current partner for, or with whom you may like to create a stable relationship, leading to cohabitation or marriage, in case of a breakup with a current partner or if you are single”; “By a short-term relationship, we mean a single date accepted on the spur of the moment, an affair within a long-term relationship, or a one-night stand.”

At the end, participants completed a questionnaire which asked about their eye color and that of their parents and current partner, if they had one. (The information on parental eye color was collected for a parallel project on parental imprinting; the corresponding data have been presented in Bressan, 2020b.) Options were “dark brown,” “brown,” “hazelnut (very light brown),” “green,” “grey,” blue,” and “other” (with the invitation to specify the exact color). Participants also reported—on a 0–3 scale from “no, never” to “yes, very often”—how much they had felt rejected by each parent during childhood via three representative items of the short form of the EMBU Rejection Scale (Arrindell et al., 1999): “I was treated as the ‘black sheep’ or ‘scapegoat’ of the family,” “She [he] would punish me hard even for small things,” and “It happened that she [he] was cold or angry with me without letting me know the reason.” The parental rejection score was the sum of the three scores, and could thus range from 0 to 9.

Finally, partnered participants were shown the photos of eight attractive male faces, presented again in identical pairs differing only in eye color (light vs dark, with light on the left half of the time). Each pair was followed by three questions. The first was, “If you suspected your partner was being unfaithful to you, which of these two men would you be more jealous of?” Possible responses were “the one on the left” and “the one on the right.” The second and third questions were, “How jealous would you be of the man on the left?” and “How jealous would you be of the man on the right?”, each followed by a 0–10 scale from “not at all jealous” to “very jealous.”

## Results

Light- and dark-eyed participants were not significantly different in any of the demographic characteristics measured in the study, such as age, ethnicity, education level, sexual orientation, partnership status, and mean partnership duration. Stimuli (i.e., face/eye-color combinations) and procedures (i.e., relationship-context order) that were counterbalanced between subjects turned out to have been presented to light- and dark-eyed participants with the same frequency. (The importance of checking the dataset for confounds related to the random assignment of participants to conditions is discussed in Bressan, 2019.)

Of the 1237 men included in the analyses, 20 (1.6%) were not Italian or raised but not born in Italy. The pattern of results remained the same if the data of these participants were discarded. Only results directly relevant to the predictions of the paternity-uncertainty hypothesis are reported here; the complete set of results can be found in the Supplementary Materials.

### Having Light Eyes Increases Men’s Preference for Light-Eyed Partners More in a Long-Term Than in a Short-Term Context

Men’s preference for light-eyed women was computed as the number of choices of light-eyed female faces, relative to the total number of choices, in response to the question “If you were looking for a long- (short-) term relationship, which of these two people would you prefer?” This index could range from 0 (light-eyed face is never chosen) to 1 (light-eyed face is always chosen).

A repeated-measures ANOVA was carried out on these preferences, with a within-subject factor of relationship context (long-term, short-term) and a between-subject factor of own eye color (light, dark). Light eyes were liked better in short- than in long-term partners, *F*(1, 1231) = 93.33, *p* < 0.0001. Light-eyed men preferred women with light eyes more than dark-eyed men did, *F*(1, 1231) = 37.30, *p* < 0.0001, η^2^_*p*_ = 0.03—an effect which is about the size of juries’ tendency to be more influenced by credible than noncredible witnesses (Richard et al., 2003). The effect was stronger when women were considered for marriage than for a one-night stand, as shown by the significant interaction between own eye color and relationship context, *F*(1, 1231) = 8.49, *p* = 0.004.

Overall, light eyes looked more attractive than dark ones (they were chosen 66% and 58% of the time by light- and dark-eyed men respectively; both *p’s* < 0.0001, one-sample *t*). This core preference for light eyes has also been found in women (Bressan & Damian, 2018) and will not be discussed here. Indeed, the point is not that *only* light-eyed men should prefer light-eyed women, but that light-eyed men should prefer light-eyed women *more* than dark-eyed men do. Whether this effect arises atop a general preference for light eyes, for dark eyes, or no preference, does not matter. The reason is that, regardless of any local baseline preference (with its attendant evolutionary benefits), choosing a light-eyed partner confers an extra selective advantage to a man if he is light-eyed but not if he is dark-eyed.

### Having Light Eyes Increases Men’s Jealousy of Light-Eyed Relative to Dark-Eyed Rivals

The effect of rivals’ light eyes on partnered men’s feelings of jealousy was computed as the number of choices of light-eyed male faces, relative to the total number of choices, in response to the question “If you suspected your partner was being unfaithful to you, which of these two men would you be more jealous of?” This index could range from 0 (light-eyed face is never chosen) to 1 (light-eyed face is always chosen).

In a potential competitor, light eyes looked more ominous than dark ones (they were selected 66% and 56% of the time by light- and dark-eyed men respectively; both *p’s* < 0.0001, one-sample *t*). These choices were analyzed with a univariate ANOVA with a fixed factor of own eye color (light, dark). Crucially, having light eyes increased the perceived threat of light-eyed rivals relative to dark-eyed ones, *F*(1, 612) = 16.62, *p* < 0.0001, η^2^_*p*_ = 0.03.

### The Effect of Having Light Eyes on Both Attraction and Jealousy Is Smaller in Men Who Felt Rejected by Their Own Fathers

Light-eyed men preferred light eyes in a potential partner more than did dark-eyed men, especially if women were imagined as long-term companions rather than one-night stands. Light-eyed men feared light eyes in a rival more than did dark-eyed men.

These results dovetail neatly in sustaining the idea that, in light-eyed men, such inclinations would have decreased paternity uncertainty. In a world of finite resources, however, choosiness is a risky luxury. Disregarding a dark-eyed woman who is available now in favor of a light-eyed woman who might or might not be available in the future amounts to the loss of a sure opportunity for an unsure one. Systematically slighting the threat of dark-eyed rivals would be a poor choice too: dark-eyed rivals are every bit as capable of breaking a relationship as are light-eyed ones. It is easy to see that indiscriminate fussiness would soon put light-eyed men at an evolutionary disadvantage relative to dark-eyed ones. There must come a point where the advantages of higher paternity confidence are surpassed by the disadvantages of lower mating opportunities. In particular, if eye-color choosiness is driven by paternity concerns and paternity concerns are driven by the prospect of paternal investment, such choosiness would be adaptive only in men who expect to invest in their children.

This raises the question of whether this particular implication of the hypothesis can be tested on the available data; that is, if any of the participants’ responses to the questionnaire could reflect the willingness to invest in one’s children. The study was designed to concurrently assess the hypothesis that people imprint on parental eye color (Bressan, 2020b). For this reason, participants reported via a compact version of the EMBU Rejection Scale (Arrindell et al., 1999) how much, as children, they had felt rejected by each parent. The literature shows that the quality of the early relationship between a man and his father predicts the level of involvement between the man and his child (e.g., Jessee & Adamsons, 2018). This might be due to the father serving as a behavioral model (Bandura, 1977), and/or to paternal acceptance having positive effects on a man’s psychological well-being and thus his relationships (Rohner & Khaleque, 2010), and/or to parenting behavior being partly heritable (Klahr & Burt, 2014). So, men who felt rejected by their own father are less likely to invest in their children than men who felt accepted by him. In an exploratory rather than confirmatory spirit, then, I reran both previous ANOVAs with paternal rejection score (split along the median, to obtain subsamples as large as possible) as an additional factor.

**Fig. 2:**
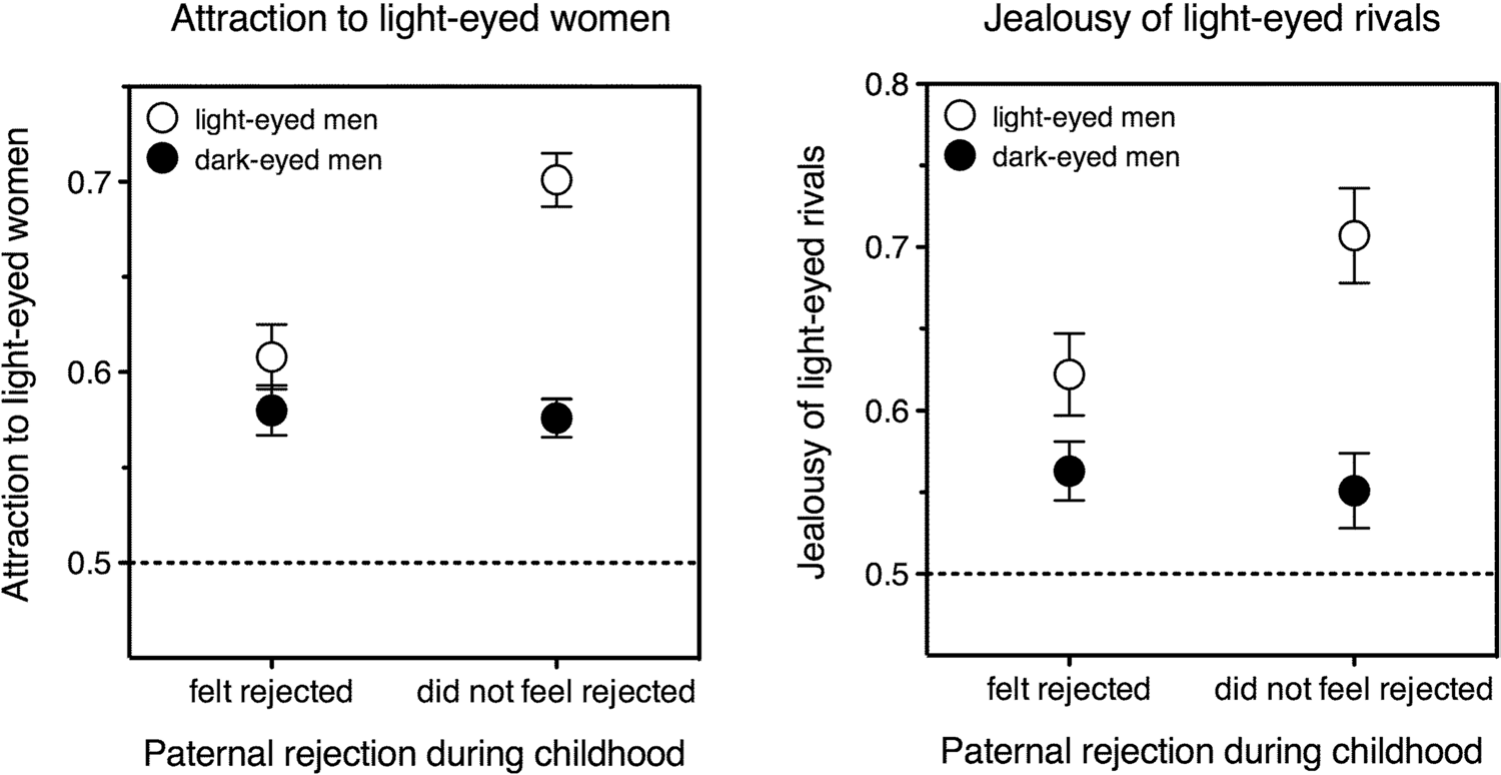
Attraction to light-eyed relative to dark-eyed partners (left panel) and jealousy of light-eyed relative to dark-eyed rivals (right panel). Attraction and jealousy are depicted as a function of having light eyes (open symbols) or dark eyes (closed symbols) and of having felt rejected (left side in each panel) or accepted (right side in each panel) by one’s father. Having light eyes increases both the attraction to light-eyed partners and the perceived threat of light-eyed rivals (in each graph, open symbols are higher up than closed symbols). However, the effect is significantly smaller for participants who reported having felt rejected by their own father while growing up (in each graph, open and closed symbols are closer together on the left than on the right side). The dotted line represents chance level (0.5); error bars indicate one standard error of the mean. Left panel, attraction question: the data refer to all the men in the sample who provided a paternal rejection rating (*N* = 1210). Right panel, jealousy question: the data refer to all the partnered men in the sample who provided a paternal rejection rating (*N* = 599). “Felt rejected”: all participants whose paternal rejection score was above the median (all men: values 2–9; partnered men: values 1–9). “Did not feel rejected”: all participants whose paternal rejection score was below the median (all men: values 0–1; partnered men: value 0)

In the repeated-measures ANOVA on men’s preferences for light eyes, paternal rejection (below the median, above the median) interacted with own eye color (light, dark), *F*(1, 1206) = 13.10, *p* = 0.0003. (Tellingly, *maternal* rejection did not: *F*(1, 1217) = 0.87, *p* = 0.350.) As shown by separate ANOVAs, having light eyes significantly increased the preference for light-eyed partners in men who had felt accepted by their father, *F*(1, 731) = 52.97, *p* < 0.0001, η^2^_*p*_ = 0.07, but not in those who had felt rejected by him, *F*(1, 475) = 1.78, *p* = 0.183 (Fig. 2, left panel). Consistently, in participants who had felt accepted by their father having light eyes was significantly more important in a long- than in a short-term context, *F*(1, 731) = 7.69, *p* = 0.006; in participants who had felt rejected it was not, *F*(1, 475) = 1.49, *p* = 0.222. A similar effect emerged in the univariate ANOVA on partnered men’s jealousy of rivals: paternal rejection (below the median, above the median) interacted with own eye color (light, dark), *F*(1, 595) = 4.09, *p* = 0.044. That is, having light eyes increased the relative threat of light-eyed rivals more in men who had felt accepted by their father than in those who had felt rejected by him (Fig. 2, right panel).

Results were similar if paternal rejection was left in its original, continuous form rather than split along the median; and if attraction and jealousy were computed not as proportion of choices, but as differences between the ratings given to light vs dark eyes on the 0–10 scale. Multiple regression analyses (Norman, 2010) showed that having light eyes increased both relative attraction to light-eyed women and relative jealousy of light-eyed rivals (own light eye color, attraction: beta = 0.225, *p* < 0.0001; jealousy: beta = 0.142, *p* = 0.007); and that the effect was dampened by paternal rejection (own light eye color × paternal rejection, attraction: beta =  −0.104, *p* = 0.014; jealousy: beta =  −0.136, *p* = 0.031).

### Light-Eyed Men’s Preference for Light-Eyed Women Is Not an Artifact of Imprinting on Parental Light Eyes

Men are attracted to potential partners whose eye color resembles their own mother’s (but not their father’s: Bressan, 2020b; Little et al., 2003), and of course light-eyed men tend to have light-eyed mothers. One could thus surmise that the current findings may be parsimoniously explained by some form of parental imprinting, with no need to resort to a hypothesis around paternity uncertainty. This possibility can be forcefully dismissed on at least two accounts. First, if this were the case, the effect of own light eyes would be smaller than that of maternal ones (since not all light-eyed men have light-eyed mothers and not all light-eyed mothers have light-eyed sons). On the contrary, it was larger, whether the effects of own and maternal eye color were considered separately (η^2^_*p*_ = 0.03 vs 0.008, that is Cohen’s *d* = 0.35 vs 0.18) or partialled out from one another. Second, as shown by separate ANOVAs, the effect of own light eyes was independently significant in men with light-eyed mothers, *F*(1, 354) = 13.75, *p* = 0.0002, and in men with dark-eyed mothers, *F*(1, 729) = 7.61, *p* = 0.006. Thus, the connection between having light eyes and liking light eyes shows up along with, not because of, sexual imprinting.

### Light-Eyed Men’s Preference for Light-Eyed Women Is Not an Artifact of Assortative Mating

People tend to be attracted to potential partners who resemble them (assortative mating), hence it is sensible to ask whether light-eyed men’s preference for light-eyed women could be a mere byproduct of this inclination. Assortative mating might be direct, by eye color, or indirect—most plausibly by ethnicity, which could create the illusion of assortative mating by eye color just because eye color and ethnicity covary. However, even disregarding the detail that all participants had the same ethnicity, neither interpretation stands up to scrutiny. First, the effect of own eye color on preference is observed for light-eyed men but not for dark-eyed ones. In Fig. 2, both light and dark symbols are above the no-preference dotted line; yet assortative mating would predict dark symbols to be below it. Second, the effect is moderated by paternal rejection, and only in light-eyed men; but one does not expect assortative mating to be affected by people’s relationship with their fathers (and if it were, not exclusively in men with light eyes).

## Discussion

Here, I have put to a triple test the idea that light-eyed men should have evolved a particular preference for light-eyed women as potential partners and a particular concern with light-eyed men as potential rivals. I have done so with the help of the data of over one thousand men who rated the facial attractiveness of potential partners, and the threat of potential rivals, whose eye color had been manipulated. On this substantial sample, I found that light-eyed men see light-eyed women as more attractive than dark-eyed men do. The effect (which extends and qualifies the results of Laeng et al., 2006) proved large, robust, and impossible to explain away by either assortative mating or imprinting on parental eye color.

I specifically tested three implications of the paternity-uncertainty account. First, the effect should be stronger when women’s attractiveness is assessed in a long-term context, that is, in a context where people expect to have children and fathers are assumed to invest in them. Second, light eyes in a rival should increase feelings of jealousy more in light-eyed than in dark-eyed men, which would prove adaptive because social fathers who have light eyes and prefer light eyes would be likelier to inappropriately invest in the offspring of light-eyed, not of dark-eyed, rivals (whereas the eye color of rivals is irrelevant for social fathers with dark eyes). Third, all these effects (light-eyed men like light-eyed partners better than dark-eyed men do; this preference is stronger in a long- than in a short-term context; light eyes in a rival increase jealousy more in light-eyed than in dark-eyed men) ought to parallel men’s expectation to invest in their children. Hence, on the grounds that men who have experienced less affective investment from their father tend to repeat this pattern with their own children (e.g., Jessee & Adamsons, 2018), these effects should diminish in men who felt rejected by their fathers. All three predictions were supported.

The roles of own eye color (light, dark), relationship context (long-term, short-term), and potential rival’s eye color (light, dark) were tested specifically by the study’s design. The role of paternal rejection was not predicted ahead of time, implying that this finding ought to be regarded as exploratory. Yet, that a man’s recollections of paternal rejection cancel, independently, all these effects suggests that such recollections do reflect a man’s disinclination to invest in his future children.

Note importantly that, via its impact on the fitness of children, paternal investment has an impact on the fitness of mothers as well. Thus, the paternal-uncertainty hypothesis by no means predicts that women should be neutral with respect to eye-color preference (indeed, they are not: Bressan & Damian, 2018), or that the preference should be the same for light- and dark-eyed women (indeed, it is not: Bressan & Damian, 2018). Yet because they demand a larger conceptual framework, predictions concerning women will be examined in a comprehensive theoretical article and are not discussed here.

Considering that proper mirrors were unavailable for most of our evolutionary history, one may wonder how people’s eye color could possibly inform their choices. One may think that the evolution of a preference for mates whose eye color looks like our own need not be more complex than that of a preference for people whose facial traits do—which has been demonstrated repeatedly and begins well before adulthood (Richter et al., 2016). Such a preference turns out to rest on information about the self rather than about one’s family members, even though the latter are usually more readily available for inspection (shown by a study on twins: Bressan & Zucchi, 2009). However, as we have seen, the preference for self-similar eye color is far more specific than a mere preference for self-similar people: most strikingly, it does not apply to dark-eyed individuals. This makes it unlikely that it could rely on mechanisms such as social mirrors, exposure to familial features, or self-contemplation on natural reflective surfaces.

It has been argued (Rantala & Marcinkowska, 2011) that a theoretical problem with the paternity-uncertainty interpretation of blue-eyed men’s preference for blue-eyed women is that the eyes of babies of European ancestry are initially “blue”—actually, slate grey—regardless of the eye color of the parents. This criticism appears misplaced, however. Paternal (unlike maternal) investment is virtually nil at childbirth and slowly increases thereafter (Geary, 2000), becoming crucial only past late childhood or adolescence (Shenk & Scelza, 2012). Yet even in societies where it is of little consequence for children’s survival, such as ours, paternal investment ends up affecting children’s success as adults a great deal (Geary, 2000; Shenk & Scelza, 2012). The few months it takes for a baby’s true eye color to reveal itself leave a father plenty of years to influence, by devoting or withholding income and time, the socioeconomic and cultural position that the child will manage to achieve in life.

## Declarations

### Availability of Data and Material

All data, analysis scripts and annotated outputs are publicly available via the Open Science Framework and can be accessed at https://osf.io/jn6xa
